# Breast Cancer Detection Patterns in Year 2 of the COVID‐19 Pandemic Highlight Gains and Gaps in Breast Cancer Surveillance

**DOI:** 10.1002/cam4.71275

**Published:** 2025-09-30

**Authors:** Susan J. Doh, Trisha Lal, Natalie Chakraborty, Uriel Kim, Richard S. Hoehn

**Affiliations:** ^1^ Division of Surgical Oncology University Hospitals Cleveland Medical Center Cleveland Ohio USA; ^2^ School of Medicine Case Western Reserve University Cleveland Ohio USA; ^3^ Department of Population and Quantitative Health Sciences, School of Medicine Case Western Reserve University Cleveland Ohio USA; ^4^ Case Comprehensive Cancer Center, School of Medicine Case Western Reserve University Cleveland Ohio USA; ^5^ Department of Internal Medicine Cedars‐Sinai Medical Center Los Angeles California USA

**Keywords:** breast cancer, COVID‐19, incidence

## Abstract

**Introduction:**

The COVID‐19 pandemic severely hindered breast cancer (BC) diagnoses. We investigated the impact of COVID‐19 on BC diagnoses in the US.

**Methods:**

We retrospectively reviewed the 2024 release of the Surveillance, Epidemiology, and End Results database (SEER: 2000–2021) for invasive BC incidences per 100,000 in adult women. Using the SEER Joinpoint software, we modeled BC incidence trends through 2019 and extrapolated expected incidences in 2020–2021. We compared expected and observed incidences and calculated percent differences (PD) and the national deficit in diagnoses.

**Results:**

Observed BC incidence in 2020 was 122.03, while expected incidence was 132.14, yielding a PD of −7.6% [−8.9%, −6.4%]. In 2021, observed BC incidence exceeded projections (3.5% [2.1%, 4.8%]). These translated to a cumulative deficit of 7190 cases [−11,886, −2494] during 2020–2021. Localized and regional BC detection decreased in 2020 and was within (1.4% [−0.9%, 3.7%]) or exceeded (4.1% [4.2%, 4.4%]) projections, respectively, in 2021. Distant disease detection was within projections in 2020 and exceeded projections in 2021 (3.9% [0.6%, 7.2%]). Across most demographic subgroups, detection was low in 2020. In 2021, cases exceeded projections in White women and metropolitan and high education/income counties, were within projections in racial/ethnic minorities and rural and low education/income counties, and were below projections in 85+ year olds and 10%–19.99% foreign‐born counties.

**Conclusions:**

BC detection recovered in 2021 compared to the start of the pandemic, but notable disparities persisted. Careful surveillance and study are needed to prevent further gaps in BC detection years after the pandemic.

## 1 Introduction

In the United States, one in eight women is diagnosed with breast cancer (BC), and one in 39 dies from BC during her lifetime [1]. Early detection is key to management [1], but the COVID‐19 pandemic disrupted BC diagnoses worldwide [2, 3]. Studies have reported increases in locally advanced and distant BC cases during the early postpandemic years [4, 5, 6, 7].

Previous US studies have explored the pandemic's impact on BC incidence as part of overall cancer detection trends [8, 9, 10]. For example, BC detection in 2020 was 8% lower than expected [8]. Overall, detection appears to have recovered in 2021 [9]. We aim to further characterize BC detection in the first 2 years of the pandemic, assess national recovery in 2021, and identify gaps in detection across clinical, demographic, and socioeconomic subgroups. We also aim to analyze trends in triple‐negative BC (TNBC), a subtype with aggressive biology and poor prognosis that disproportionately affects young and non‐Hispanic Black women [11].

## 2 Methods

### 2.1 Data Source and Institutional Review Board (IRB) Exemption

This is a retrospective study of the Surveillance, Epidemiology, and End Results database (SEER), which covers 48% of the U.S. population and is deidentified and publicly available at www.seer.cancer.gov with the SEER*Stat Database. Our study was exempted by the University Hospitals Cleveland Medical Center IRB (STUDY20240595).

BC incidence data were abstracted from the April 2024 release of SEER based on the November 2023 Submission accessed July 2024 [12], and we utilized delay adjustment where possible (https://surveillance.cancer.gov/delay/). As previously described [8], using the SEER Joinpoint Regression Program (Version 5.1.0.0, April 2024, Statistical Research and Applications Branch, National Cancer Institute), we modeled BC incidence trends from 2000 to 2019 using permutation tests [13] and projected expected incidences for 2020 and 2021. We calculated percent differences (PD) between expected and observed incidence rates during 2020 and 2021 to assess the “disruption” and subsequent “recovery” in BC detection. Statistical tests were conducted at *α* = 0.05. With estimation of 95% confidence intervals (CI) of incidence rates and PDs, we calculated the national deficit in detected cases as previously described [14].

## 3 Results

Observed BC incidence in 2020 was 122.03 per 100,000 women versus the expected 132.14, resulting in a significant PD of −7.6% [95% CI: −8.9%, −6.4%]. In 2021, observed BC incidence exceeded projections, with a statistically significant positive PD of 3.5% [2.1%, 4.8%] (Table 1). This translated to 16,860 undetected cases in 2020 but 9671 additional diagnoses in 2021, yielding a cumulative deficit of 7190 cases [−11,886, −2494] across 2020 and 2021 (Figure S1).

**TABLE 1:** Disruption and recovery of breast cancer detection during Years 1 and 2 of the pandemic.

	Pandemic Year 1 (2020)				Pandemic Year 2 (2021)			
	Incidence per 100,000				Incidence per 100,000			
	Expected	Observed	Percent difference	95% CI (%)	Expected	Observed	Percent difference	95% CI (%)
All breast cancer cases	132.14	122.03	**−7.6**	**−8.9 to −6.4**	132.80	137.42	**3.5**	**2.1 to 4.9**
Localized	86.25	77.21	**−10.5**	**−12.6 to −8.4**	87.16	88.37	1.4	−0.9 to 3.7
Regional	33.91	32.54	**−4.0**	**−4.1 to −4.0**	33.64	35.08	**4.3**	**4.2 to 4.4**
Distant	7.70	7.55	−1.9	−5.1 to 1.3	7.76	8.06	**3.9**	**0.6 to 7.2**
Receptor subtypes								
HR+/HER2—	94.60	84.80	**−10.4**	**−13.8 to −7.0**	96.55	97.02	0.5	−3.3 to 4.2
HR‐/HER2+	4.86	4.88	0.5	−1.7 to 2.6	4.73	5.59	**18.2**	**15.5 to 20.9**
HR+/HER2+	12.20	11.71	**−4.1**	**−6.3 to −1.8**	11.88	12.56	**5.8**	**3.2 to 8.3**
HR‐/HER2—	13.57	13.02	**−4.1**	**−6.5 to −1.7**	13.63	14.53	**6.6**	**4.0 to 9.2**
Race/ethnicity								
Hispanic (any race)	107.61	96.08	**−10.7**	**−14.7 to −6.7**	109.25	111.07	1.7	−2.8 to 6.1
NH AI/AN	126.21	104.39	**−17.3**	**−29.0 to −5.6**	128.57	131.01	1.9	−11.5 to 15.3
NH Asian/PI	116.86	102.45	**−12.3**	**−17.2 to −7.5**	119.24	125.52	5.3	−0.4 to 10.9
NH Black	135.04	122.89	**−9.0**	**−11.6 to −6.4**	136.01	135.58	−0.3	−3.1 to 2.4
NH White	141.82	131.69	**−7.1**	**−8.7 to −5.6**	142.54	146.58	**2.8**	**1.2 to 4.5**
Age group (years)								
< 20	0.06	0.046	−25.1	−74.5 to 24.2	0.06	0.048	−22.3	−73.6 to 28.9
20–39	30.22	29.09	−3.7	−8.7 to 1.3	30.57	31.35	2.5	−2.7 to 7.8
40–54	192.03	179.08	**−6.7**	**−10.2 to −3.3**	194.21	200.14	3.1	−0.7 to 6.8
55–69	342.54	313.06	**−8.6**	**−9.9 to −7.3**	343.41	350.25	**2.0**	**0.6 to 3.4**
70–84	476.78	423.58	**−11.2**	**−13.3 to −9.0**	481.92	489.31	1.7	−0.6 to 4.1
85+	361.52	305.11	**−15.6**	**−21.6 to −9.6**	373.30	341.49	**−8.5**	**−14.9 to −2.2**
County characteristics								
Rurality								
Large Metropolitan	132.69	120.57	**−9.1**	**−10.4 to −7.9**	133.20	136.61	**2.6**	**1.2 to 3.9**
Medium Metropolitan	130.77	121.14	**−7.4**	**−9.4 to −5.3**	131.42	133.67	1.7	−0.4 to 3.9
Small Metropolitan	132.86	123.17	**−7.3**	**−11.4 to −3.2**	134.35	132.43	−1.4	−5.7 to 2.9
Rural, adjacent to metropolitan area	126.52	118.36	**−6.4**	**−10.2 to −2.7**	127.74	123.70	−3.2	−7.0 to 0.7
Rural, not adjacent to metropolitan area	119.17	109.74	**−7.9**	−**12.1 to −3.8**	119.92	115.15	−4.0	−8.2 to 0.2
Poverty								
< 10%	138.84	127.96	**−7.8**	**−9.3 to −6.3**	139.41	144.72	**3.8**	**2.2 to 5.4**
10%–19.99%	128.44	117.36	**−8.6**	**−9.9 to −7.3**	129.02	130.31	1.0	−0.4 to 2.4
20%+	117.57	108.56	**−7.7**	**−10.6 to −4.7**	118.33	116.45	−1.6	−4.7 to 1.5
% Foreign Born								
< 10%	132.08	124.05	**−6.1**	**−8.1 to −4.1**	132.98	133.28	0.2	−1.9 to 2.3
10%–19.99%	141.25	125.27	**−11.3**	**−13.9 to −8.7**	144.21	138.83	**−3.7**	**−6.5 to −1.0**
20%+	128.20	115.13	**−10.2**	**−11.8 to −8.6**	128.61	132.35	**2.9**	**1.1 to 4.7**
% Without high school diploma								
< 10%	140.77	129.63	**−7.9**	**−9.5 to −6.3**	141.48	144.39	**2.1**	**0.3 to 3.8**
10%–19.99%	126.96	116.04	**−8.6**	**−10.1 to −7.1**	127.45	130.12	**2.1**	**0.5 to 3.7**
20%+	113.14	103.53	**−8.5**	**−11.6 to −5.3**	113.89	111.46	−2.1	−5.4 to 1.1

*Note:* Bolded figures indicate statistically significant (*p* < 0.05) values.

Abbreviations: AI/AN, American Indian/Alaska Native; CI, confidence interval; NH, non‐Hispanic; PI, Pacific Islander.

By cancer stage (Table 1), localized BC detection decreased in 2020 (−10.5% [−12.6%, −8.4%]) but was not statistically significantly different from expected levels in 2021 (1.4% [−0.9%, 3.7%]). Regional BC decreased in 2020 (−4.0% [−4.1%, −4.0%]) but exceeded projections in 2021 (4.1% [4.2%, 4.4%]). Distant BC detection was within projections in 2020 but was statistically significantly increased in 2021 (3.9% [0.6%, 7.2%]).

Among receptor subtypes, hormone receptor positive (HR+) human epidermal growth factor receptor 2 negative (HER2‐) BC detection decreased the most in 2020 (−10.4% [−13.8%, −7.0%]) but was within expected projections in 2021 (0.5%, [−3.3%, 4.2]). HR+/HER2+ and HR‐/HER2‐ BC detection decreased in 2020 (−4.1% [−6.3%, −1.8%] and −4.1% [−6.5%, −1.7%], respectively) and exceeded projections in 2021 (5.8% [3.2%, 8.3%] and 6.6% [4.0%, 9.2%], respectively).

Most demographic subgroups recovered by 2021. BC incidence in non‐Hispanic White women (2.8% [1.2%, 4.5%]), large metropolitan areas (2.6% [1.2%, 3.9%]), 20%+ foreign‐born counties (2.9% [1.1%, 4.7%]), low poverty counties (3.8% [2.2%, 5.4%]), and high‐education counties (2.1% [0.3%, 3.8%]) exceeded projections in 2021. In contrast, BC detection in women of racial minorities in 2021 recovered but did not exceed projections: Hispanic: 1.7% [−2.8%, 6.1%], American Indian/Alaska Native: 1.9% [−11.5%, 15.3%], Asian and Pacific Islander: 5.3% [−0.4%, 10.9%], and non‐Hispanic Black women: −0.3% [−3.1%, 2.4%]. This was also true for women residing in counties of low income (−1.6% [−4.7%, 1.5%]), rural communities (−4.0% [−8.2%, 0.2%]), and counties of low education (−2.1% [−5.4%, 1.1%]) in 2021. Persistent deficits into 2021 were observed in women aged 85+ (−8.5% [−14.9%, −2.2%]) and women in 10%–19.99% foreign‐born counties (−3.7% [−6.5%, −1.0%]).

Subgroup analysis of TNBC (Table S1) showed localized TNBC detection decreased in 2020 (−6.1% [−9.1%, −3.0%]) but exceeded projections in 2021 (5.1% [1.9%, 8.4%]). Regional TNBC incidence was within expected levels during 2020 (−1.5% [−4.3%, 1.3%]) and increased in 2021 (5.7% [2.8%, 8.6%]). Most demographic subgroups' BC incidences recovered by 2021.

## 4 Discussion

BC detection did not reach prepandemic levels in 2020, as confirmed by decreased BC screening [15, 16, 17, 18, 19, 20] and biopsy rates [16, 21]. Consistent with previous findings [9], BC incidence recovered in 2021. Nonnegative PDs likely reflect decreased barriers and women's abilities to resume screening and diagnostic services after the acute pandemic period. As indicated by the 3.5% positive PD of overall BC cases in 2021, women reengaged with healthcare systems after the pandemic in 2020. Correspondingly, screening mammography volumes recovered in 2021 [22, 23, 24], along with BC diagnoses [24].

Regional and distant BC detection increased by 4.3% and 3.9%, respectively, in 2021. In contrast, a SEER study reported increased distant disease detection in 2021 [9]. Delays in BC detection in 2020 may have contributed to BC progression to advanced stages in 2021. However, BC grows slowly, with doubling times averaging 212 days to 2.1 years [25, 26, 27, 28, 29]. Alternatively, symptomatic women with advanced disease seeking care in 2021 increased as pandemic‐related barriers eased. Detection delays could allow tumors to progress in tumor‐ or nodal‐stages, heightening recurrence risks later [30]. TNBC trends also underscore these future recurrence risks. Localized, but not regional, TNBC detection decreased in 2020, while regional disease increased in 2021. The aggressive biology and faster growth rates of TNBC [28, 31] suggest diagnostic delays in 2020 led to progression to advanced disease by 2021. Long‐term monitoring is necessary to fully assess the impact of these delays on BC recurrence.

Disparities in recovery were evident. In 2020, all demographic subgroups experienced decreased BC detection. In 2021, White women experienced a rebound in detection with a positive PD of 2.8%. Studies have shown greater return to mammographic screening in 2021 in suburban hospitals, which served predominantly White patients [23] and in White patients overall after the acute pandemic period [32]. In contrast, BC detection in Hispanic, American Indian/Alaska Native (AIAN), Asian, Pacific Islander, and Black women recovered to incidences within prepandemic projections. This is consistent with greater reductions in screening in minority women during the pandemic [20] and lower screening rates in early reopening months [33] and postpandemic [32]. Black and Hispanic women face unique barriers, including reduced access to accredited breast imaging centers of excellence [34], which contribute to lower BC screening utilization rates than White women [35]. Black and Hispanic women are also more likely to be diagnosed younger, with more advanced disease and aggressive subtypes like TNBC [36]. Furthermore, higher age‐adjusted incidence rates of COVID‐19‐related hospitalizations among patients of Hispanic, Black, and AIAN races in 2020 [37] and 2021 [38] may have hindered BC screenings in 2021. Similarly, barriers to screening existed in rural communities, higher poverty areas, and lower education counties during the pandemic [39, 40] and undoubtedly persisted into 2021.

Women aged 85+ and those in counties with 10%–19.99% foreign‐born populations experienced persistent detection deficits in 2021. BC detection in 2021 for women aged 85+ decreased by −8.5%. The wide confidence interval (−14.9% to −2.2%) is likely due to the smaller population of this age group, resulting in greater uncertainty in true population parameters. Nevertheless, elderly women likely faced challenges for BC diagnosis due to increased COVID‐19 morbidity [41]. In our study, BC incidence decreased at −3.7% in 2021 in 10%–19.99% foreign‐born counties but not in > 20% foreign‐born counties. The lack of census‐level granularity may account for this mixed finding. Cancer screening adherence in US foreign‐born populations is modified by a complex web of factors, including healthcare access, cultural beliefs, limited English language proficiency and interpreter availability, patient‐provider racial/ethnic concordance, and patient‐provider language concordance [42, 43, 44, 45, 46]. Proactive measures are needed to further address barriers in the postpandemic period.

A major limitation of this study is that SEER does not cover 100% of the population. Additionally, this study did not assess specific barriers to BC detection in 2021, limiting our ability to determine why certain communities experienced slower recoveries postpandemic. Nevertheless, our findings highlight critical gaps in BC detection during the postpandemic period and serve as a basis for generating hypotheses. Further efforts by hospitals and local healthcare systems are essential to address barriers exacerbated by the pandemic. Barriers that hindered breast cancer screening and diagnosis during the pandemic—and continue to persist—must be identified and mitigated.

## 5 Conclusion

BC detection efforts resumed in 2021, but recovery was uneven, with certain groups recovering to a lesser degree or not recovering altogether. This was seen in vulnerable populations, including the elderly, rural residents, immigrants, and racial minorities, as well as low socioeconomic groups. To mitigate disparities in BC detection and outcomes in the postpandemic period, healthcare systems must address barriers to screening. Barriers to posttreatment surveillance for women who may have presented with more advanced‐stage disease in 2021 due to pandemic‐related delays must be addressed as well. Maintaining robust epidemiological surveillance is essential to understanding and addressing these inequities in the years ahead.

## Author Contributions

**Susan J. Doh:** formal analysis (equal), validation (equal), writing – original draft (lead), writing – review and editing (equal). **Trisha Lal:** writing – original draft (supporting), writing – review and editing (supporting). **Natalie Chakraborty:** writing – original draft (supporting), writing – review and editing (supporting). **Uriel Kim:** conceptualization (equal), formal analysis (equal), methodology (lead), project administration (equal), supervision (equal), writing – review and editing (equal). **Richard S. Hoehn:** conceptualization (equal), project administration (equal), supervision (equal), writing – review and editing (equal).

## Disclosure

All authors have read and agreed to the published version of the manuscript. The authors have no disclosures that impacted this work.

## Conflicts of Interest

The authors declare no conflicts of interest.

## Supporting information


**Figure S1:** cam471275‐sup‐0001‐FigureS1.docx.

**Table S1:** cam471275‐sup‐0002‐TableS1.docx.

## Data Availability

The data used is publicly available and deidentified and available at www.seer.cancer.gov with the SEER*Stat Database.
