# Supplementary material for: Breast Cancer Detection Patterns in Year 2 of the COVID‐19 Pandemic Highlight Gains and Gaps in Breast Cancer Surveillance
Source: Cancer Med. 2025 Sep 30;14(19):e71275. doi: 10.1002/cam4.71275 (PMC12483837; doi:10.1002/cam4.71275)

**Supplemental Figure 1.** Trends in Breast Cancer Incidence and Total Cases Before and After the COVID-19 Pandemic


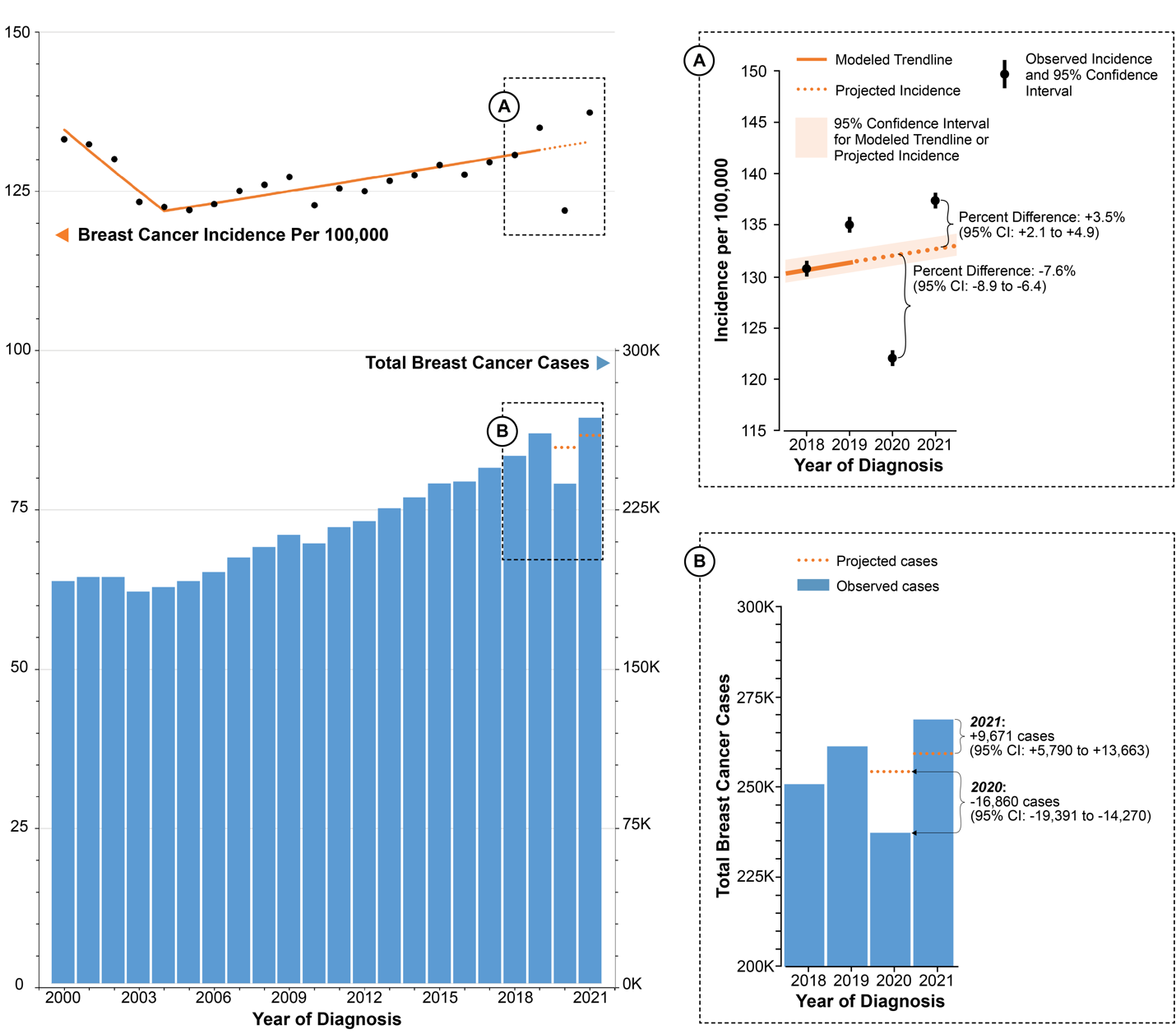

Supplement: Supplementary file 1 — Figure S1: cam471275‐sup‐0001‐FigureS1.docx. [file CAM4-14-e71275-s001.docx]
