# Supplementary material for: Breast Cancer Detection Patterns in Year 2 of the COVID‐19 Pandemic Highlight Gains and Gaps in Breast Cancer Surveillance
Source: Cancer Med. 2025 Sep 30;14(19):e71275. doi: 10.1002/cam4.71275 (PMC12483837; doi:10.1002/cam4.71275)
Supplement: Supplementary file 2 — Table S1: cam471275‐sup‐0002‐TableS1.docx. [file CAM4-14-e71275-s002.docx]

**Supplemental Table 1**. Disruption and Recovery of Triple Negative Breast Cancer Detection during Year 1 and 2 of the Pandemic

|  | | **Pandemic Year 1 (2020)** | | | | **Pandemic Year 2 (2021)** | | | |
| --- | --- | --- | --- | --- | --- | --- | --- | --- | --- |
|  | | *Incidence per 100,000* | | | | *Incidence per 100,000* | | | |
|  | | Expected | Observed | Percent Difference | 95% CI (%) | Expected | Observed | Percent Difference | 95% CI (%) |
| All Triple Negative Breast cancers | | 13.57 | 13.02 | **-4.1** | **-6.5 to -1.7** | 13.63 | 14.53 | **6.6** | **4.0 to 9.2** |
| Localized | | 8.23 | 7.73 | **-6.1** | **-9.1 to -3.0** | 8.28 | 8.70 | **5.1** | **1.9 to 8.4** |
| Regional | | 4.16 | 4.10 | -1.5 | -4.3 to 1.3 | 4.15 | 4.38 | **5.7** | **2.8 to 8.6** |
| Distant | | 1.14 | 0.99 | -13.6 | -29.9 to 2.7 | 1.25 | 1.11 | -10.6 | -27.1 to 5.9 |
| Race/Ethnicity | |  |  |  |  |  |  |  |  |
| Hispanic (any race) | | 11.79 | 11.38 | -3.5 | -10.4 to 3.4 | 11.99 | 12.63 | 5.3 | -1.9 to 12.6 |
| NH AI / AN | | 15.47 | 10.73 | -30.6 | -70.3 to 9.0 | 17.85 | 12.78 | -28.4 | -68.0 to 11.2 |
| NH Asian / PI | | 9.67 | 8.86 | -8.4 | -16.7 to 0.0 | 9.80 | 11.14 | **13.7** | **4.3 to 23.1** |
| NH Black | | 25.26 | 23.76 | **-6.0** | **-11.3 to -0.6** | 25.54 | 25.76 | 0.9 | -4.7 to 6.4 |
| NH White | | 12.48 | 11.93 | **-4.4** | **-6.8 to -2.1** | 12.47 | 13.32 | **6.8** | **4.3 to 9.3** |
| Age Group | |  |  |  |  |  |  |  |  |
| 20-39 | | 5.39 | 5.61 | 4.1 | -4.1 to 12.3 | 5.46 | 5.81 | 6.4 | -1.8 to 14.7 |
| 40-54 | | 20.78 | 20.30 | -2.3 | -5.5 to 0.8 | 20.74 | 22.22 | 7.2 | 3.9 to 10.5 |
| 55-69 | | 34.69 | 31.88 | **-8.1** | **-14.3 to -1.9** | 35.47 | 34.95 | -1.5 | -8.0 to 5.1 |
| 70-84 | | 40.93 | 36.05 | **-11.9** | **-17.1 to -6.7** | 41.49 | 43.17 | 4.0 | -1.7 to 9.8 |
| 85+ | | 27.12 | 25.61 | -5.6 | -15.1 to 3.9 | 27.34 | 33.31 | **21.8** | **10.8 to 32.9** |
| County Characteristics | |  |  |  |  |  |  |  |  |
| Rurality | |  |  |  |  |  |  |  |  |
| Large Metropolitan | | 13.42 | 12.93 | **-3.7** | **-6.3 to -1.0** | 13.46 | 14.31 | **6.3** | **3.5 to 9.1** |
| Medium Metropolitan | | 13.73 | 12.96 | **-5.6** | **-10.2 to -1.0** | 13.77 | 14.64 | **6.3** | **1.4 to 11.2** |
| Small Metropolitan | | 15.22 | 13.47 | **-11.5** | **-21.9 to -1.1** | 15.58 | 15.26 | -2.1 | -13.1 to 9.0 |
| Rural, adjacent to metropolitan area | | 14.07 | 13.10 | -6.9 | -15.4 to 1.6 | 14.21 | 13.96 | -1.7 | -10.4 to 6.9 |
| Rural, not adjacent to metropolitan area | | 12.53 | 12.21 | -2.5 | -12.8 to 7.8 | 12.54 | 12.59 | 0.5 | -9.9 to 10.8 |
| Poverty | |  |  |  |  |  |  |  |  |
| <10% | | 12.73 | 12.65 | -0.6 | -4.0 to 2.9 | 12.75 | 13.96 | **9.5** | **5.9 to 13.1** |
| 10-19.99% | | 13.90 | 12.88 | **-7.3** | **-10.3 to -4.3** | 13.98 | 14.43 | 3.2 | 0.0 to 6.4 |
| 20%+ | | 14.93 | 15.20 | 1.9 | -5.8 to 9.5 | 15.00 | 15.59 | 4.0 | -3.7 to 11.7 |
| % Foreign Born | |  |  |  |  |  |  |  |  |
| <10% | | 14.38 | 13.75 | **-4.4** | **-8.5 to -0.3** | 14.47 | 14.96 | 3.4 | -0.9 to 7.7 |
| 10-19.99% | | 13.74 | 13.42 | -2.3 | -7.1 to 2.4 | 13.84 | 14.73 | **6.4** | **1.4 to 11.4** |
| 20%+ | | 12.96 | 12.18 | **-6.0** | **-8.8 to -3.2** | 12.97 | 13.75 | **6.0** | **3.0 to 8.9** |
| % No High School Education | |  |  |  |  |  |  |  |  |
| <10% | | 13.90 | 12.97 | **-6.7** | **-11.4 to -2.0** | 14.04 | 14.55 | 3.6 | -1.4 to 8.6 |
| 10-19.99% | | 13.62 | 12.93 | **-5.1** | **-8.3 to -1.9** | 13.71 | 14.34 | **4.6** | **1.2 to 8.1** |
| 20%+ | | 13.39 | 12.99 | -3.0 | -10.7 to 4.7 | 13.45 | 13.19 | -1.9 | -9.7 to 5.8 |
|  | NH: Non-Hispanic  AI / AN: American Indian / Alaska Native  PI: Pacific Islander  CI: Confidence Interval  **Bolded** figures indicate statistically significant (p<0.05) values | | | | | | | |  |
